# Disrupted Maturation of the Microbiota and Metabolome among Extremely Preterm Infants with Postnatal Growth Failure

**DOI:** 10.1038/s41598-019-44547-y

**Published:** 2019-06-03

**Authors:** Noelle E. Younge, Christopher B. Newgard, C. Michael Cotten, Ronald N. Goldberg, Michael J. Muehlbauer, James R. Bain, Robert D. Stevens, Thomas M. O’Connell, John F. Rawls, Patrick C. Seed, Patricia L. Ashley

**Affiliations:** 1 Division of Neonatology, Department of Pediatrics, Duke University, Durham, NC 27710 USA; 2 Duke Molecular Physiology Institute and Sarah W. Stedman Nutrition and Metabolism Center, Duke University, Durham, NC 27701 USA; 3 Department of Pharmacology and Cancer Biology, Duke University, Durham, NC 27710 USA; 4 Division of Endocrinology and Metabolism, Department of Medicine, Duke University, Durham, NC 27710 USA; 5 Department of Otolaryngology, Head and Neck Surgery, Indiana University, Indianapolis, IN 46202 USA; 6 Department of Molecular Genetics and Microbiology, Duke University, Durham, NC 27710 USA; 7 Department of Pediatrics, Northwestern University, Chicago, IL 60611 USA

**Keywords:** Microbiome, Metabolomics, Paediatric research

## Abstract

Growth failure during infancy is a major global problem that has adverse effects on long-term health and neurodevelopment. Preterm infants are disproportionately affected by growth failure and its effects. Herein we found that extremely preterm infants with postnatal growth failure have disrupted maturation of the intestinal microbiota, characterized by persistently low diversity, dominance of pathogenic bacteria within the *Enterobacteriaceae* family, and a paucity of strictly anaerobic taxa including *Veillonella* relative to infants with appropriate postnatal growth. Metabolomic profiling of infants with growth failure demonstrated elevated serum acylcarnitines, fatty acids, and other byproducts of lipolysis and fatty acid oxidation. Machine learning algorithms for normal maturation of the microbiota and metabolome among infants with appropriate growth revealed a pattern of delayed maturation of the microbiota and metabolome among infants with growth failure. Collectively, we identified novel microbial and metabolic features of growth failure in preterm infants and potentially modifiable targets for intervention.

## Introduction

Postnatal growth failure is a pervasive problem among preterm infants that occurs during a critical developmental period. Previous studies have shown that the extrauterine growth rates of extremely preterm (EPT) infants (*i*.*e*., birth gestational age ≤27 weeks) almost universally fall below reference *in utero* growth rates, and approximately half of infants remain below the 10^th^ percentile in weight at the time of neonatal intensive care unit (NICU) discharge^1,2^. Compared with full term infants, preterm infants have lower weight, length, and lean body mass at full term-equivalent age, but higher percent body fat^3^. Poor growth in the NICU is a risk factor for poor neurodevelopmental outcomes, including cognitive and motor impairment, and the metabolic adaptations associated with early life growth failure may predispose preterm infants to obesity and other adverse cardiometabolic outcomes in later life^4–6^.

The etiology of postnatal growth failure in EPT infants is complex and multifactorial. Major contributing factors include inadequate nutrition, immaturity of the digestive and metabolic systems, high metabolic demands, and critical illness^7,8^. Previous research has focused on preventing and correcting nutritional deficits in preterm infants, leading to widespread adoption of proactive nutritional practices in the NICU, including early provision of parenteral and fortified enteral nutrition^9^. However, many infants continue to experience growth failure despite modern nutritional management^2^.

The intestinal microbiota has an integral role in nutrient utilization and the regulation of host metabolism. Previous human and animal studies have identified microbial and metabolic signatures associated with obesity and insulin resistance^10–12^. Recent studies have demonstrated that childhood malnutrition is associated with persistent immaturity of the gut microbiota^13–15^. Transplantation of the microbiota of malnourished children to germ-free animals transmits a phenotype of impaired growth and metabolism, indicating a causal relationship between the microbiota and malnutrition^15^. The extent to which the intestinal microbiota influences metabolism and growth in preterm infants is unknown. The early life experiences of EPT infants are vastly different from full term infants in terms of medical treatments, diet, and environmental exposures. Further, microbial colonization of the EPT infant occurs at a much earlier stage of intestinal and immune development. Studies have shown stark differences in the microbiota composition of preterm infants compared to full term infants^16,17^. There are a number of potential mechanisms by which the altered microbiota and the bioactive metabolites produced by microbial metabolism may affect growth and metabolism in preterm infants, including direct effects on nutrient acquisition and utilization as well as indirect effects on intestinal development, inflammation, and hormonal signaling^18–21^.

To investigate the relationships between the intestinal microbiota, host metabolism, and growth, we conducted a longitudinal, prospective cohort study of EPT infants throughout their NICU hospitalization. We hypothesized that the diagnosis of severe postnatal growth failure would be preceded by perturbations in the development of the microbiota and host metabolome.

## Results

We enrolled 60 EPT infants with a median birth gestational age of 26 (IQR: 24–27) weeks and birth weight of 800 (IQR: 658–895) grams. When the infants reached 40 weeks’ postmenstrual age (PMA; defined as birth gestational age plus chronologic age) or hospital disposition, 36 (60%) infants had severe postnatal growth failure (defined as weight less than the third percentile on sex-specific Fenton growth charts) and 22 (37%) infants had appropriate growth^22^. Two (3%) infants died prior to 40 weeks’ PMA and were excluded from the analysis, resulting in a cohort of 58 infants for analysis. Infants with growth failure had lower birth gestational age, lower birth weight, and required mechanical ventilation for longer than infants with appropriate growth (Table 1). All infants received parenteral nutrition immediately following birth; enteral feedings were initiated and advanced in volume and caloric content according to a feeding protocol (Table S1). All infants received human milk as their initial diet. At 40 weeks’ PMA or disposition, infants with growth failure had significantly lower percentiles for weight, length, and head circumference than infants with appropriate growth.

**Table 1:** Clinical Characteristics.

	All Infants		Infants without Sepsis, Necrotizing Enterocolitis, or Intestinal Perforation	
	Appropriate Growth (n = 22)	Growth Failure (n = 36)	Appropriate Growth (n = 20)	Growth Failure (n = 21)
Gestational age (wks), med (IQR)	27 (26, 27)	25 (24, 26)*	27 (26, 27)	26 (25, 27)*
Birth weight (g), med (IQR)	873 (816, 1063)	753 (640, 845)*	925 (820, 1073)	755 (640, 860)*
Race, n (%)				
Asian	3 (14)	3 (8)	3 (15)	1 (5)
Black or African American	13 (59)	16 (44)	11 (55)	10 (48)
White	5 (23)	17 (47)	5 (25)	10 (48)
Unknown or not reported	1 (5)	0 (0)	1 (5)	0 (0)
Female sex, n (%)	12 (55)	18 (50)	10 (50)	10 (48)
Multiple gestation, n (%)	8 (36)	9 (25)	8 (40)	5 (24)
Antenatal steroids, n (%)	22 (100)	34 (94)	20 (100)	20 (95)
Antenatal antibiotics, n (%)	16 (73)	24 (67)	15 (75)	12 (57)
C-section delivery, n (%)	17 (77)	27 (75)	15 (75)	15 (71)
**Growth outcomes**				
Weight percentile, med (IQR)	10 (6, 14)	<1 (<1, 1)*	10 (7, 16)	<1 (<1, 1)*
Length percentile, med (IQR)	7 (2, 11)	<1 (<1, <1)*	7 (3, 11)	<1 (<1, <1)*
Head circumference, med (IQR)	25 (9, 41)	2 (<1, 6)*	26 (15, 44)	2 (<1, 6)*
**Morbidities and Therapies**				
Late-onset sepsis, n (%)	1 (5)	6 (17)	0 (0)	0 (0)
Spontaneous intestinal perforation, n (%)	0 (0)	6 (17)	0 (0)	0 (0)
Medical necrotizing enterocolitis, n (%)	1 (5)	3 (8)	0 (0)	0 (0)
Surgical necrotizing enterocolitis, n (%)	0 (0)	4 (11)	0 (0)	0 (0)
Severe intraventricular hemorrhage, n (%)	2 (9)	3 (8)	2 (10)	1 (5)
Severe retinopathy of prematurity, n (%)	2 (9)	13 (36)*	2 (10)	7 (33)
Ligation of patent ductus arteriosus, n (%)	2 (9)	9 (25)	2 (10)	4 (19)
First day enteral feeds, med (IQR)	4 (3, 5)	5 (4, 9)	4 (3, 5)	5 (3, 10)
Day of first full feeds sample collection, med (IQR)	28 (20, 38)	42 (30, 55)*	26 (19, 38)	35 (25, 42)
Initial days antibiotics, med (IQR)	2 (2, 7)	2 (2, 6)	2 (2, 7)	2 (2, 6)
Total days antibiotics, med (IQR)	15 (8, 20)	20 (12, 37)	12 (8, 19)	14 (11, 25)
Initial days mechanical ventilation, med (IQR)	3 (1, 6)	9 (2, 19)*	3 (1, 5)	11 (2, 18)*
Total days mechanical ventilation, med (IQR)	4 (2, 12)	19 (8, 27)*	3 (2, 11)	16 (4, 22)*

Continuous variables compared by Wilcoxon rank-sum test and categorical variables by Fisher’s exact test. *p < 0.05.

Many infants in the cohort experienced medical complications of extreme prematurity, including late-onset sepsis (defined as culture-proven sepsis that occurred after the first 72 hours of life), necrotizing enterocolitis, and spontaneous intestinal perforation (Table 1). Infants with one or more of these complications were more likely to have growth failure than infants without these complications (88% vs. 51%, p = 0.01), and had a median weight percentile less than the first percentile at 40 weeks’ PMA. Given the potential confounding effects of these morbidities and their treatments, we conducted a secondary analysis in which we excluded infants with late-onset sepsis, medical or surgical necrotizing enterocolitis, and spontaneous intestinal perforation. A total of 41 infants remained in the secondary analysis, including 21 infants with growth failure and 20 infants with appropriate growth. There were no significant differences in the timing of enteral feeding initiation or in the number of days of antibiotics between the infants with growth failure and infants with appropriate growth (Table 1).

### Growth failure is associated with altered composition and diversity of the microbiota

**Figure 1:**
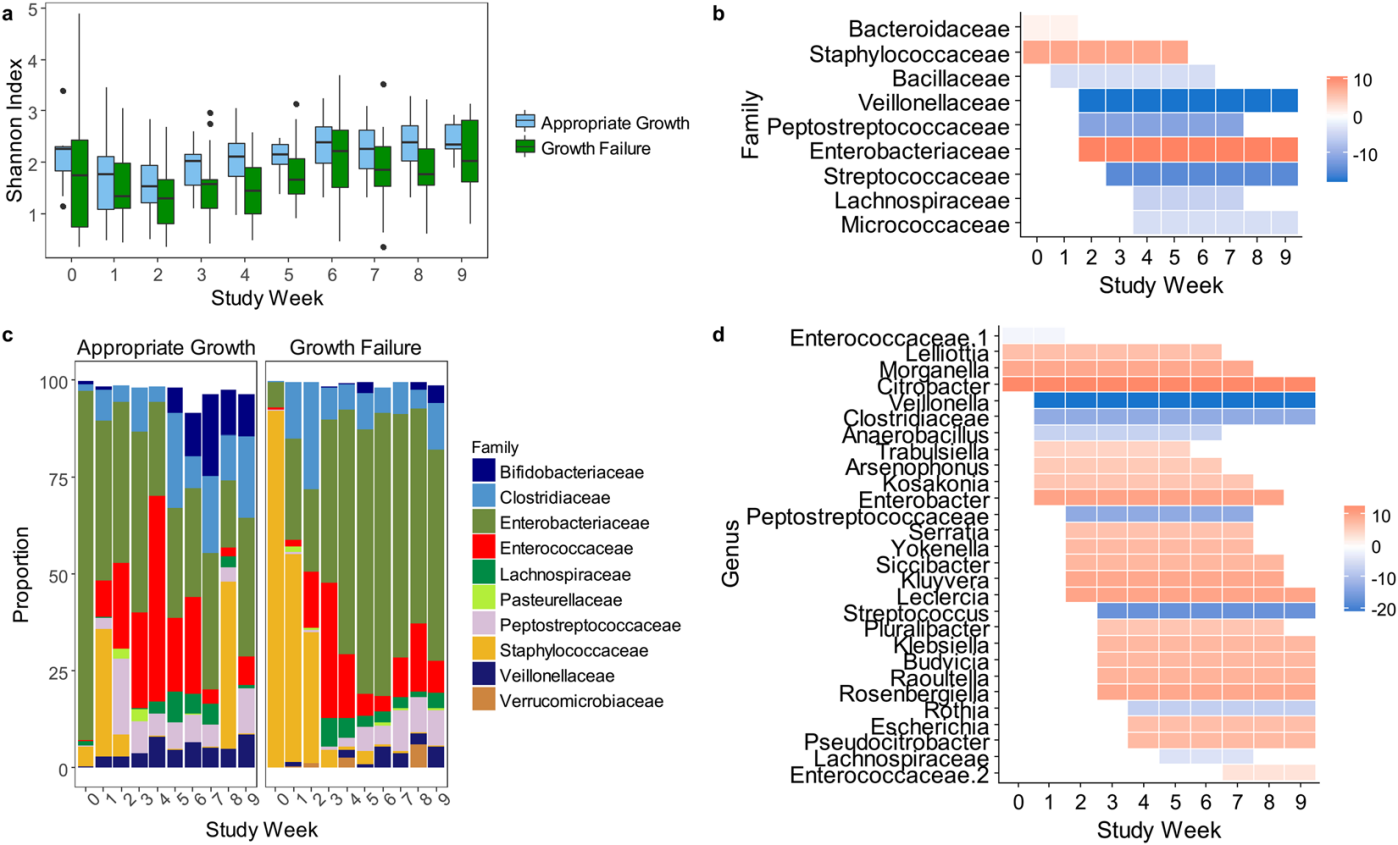
Microbiota diversity and composition. (**a**) Shannon diversity was higher over weeks 1–9 in appropriate growth (blue) vs. growth failure (green) (compared by SS-ANOVA with study week as continuous variable, p = 0.002). (**b**) Bacterial families with significant differences in relative abundance between groups by SS-ANOVA. The shaded areas represent the time intervals over which the abundance was higher in appropriate growth (blue) or growth failure (red). (**c**) Relative abundance of the top 10 bacterial families over time. (**d**) Bacterial genera with higher relative abundance in appropriate growth (blue) or growth failure (red) by SS-ANOVA. AG, appropriate growth. GF, growth failure.

We used 16S rRNA gene sequencing to compare the fecal microbiota of infants with growth failure and appropriate growth using samples collected in the first postnatal week (study week 0) and weekly for up to 9 weeks once the infants reached full enteral feedings (study weeks 1–9). Two time variables were used to measure temporal changes: study week was used to provide a consistent measure of the time elapsed since the infant reached full enteral feedings (study week 1), ensuring similar nutritional exposures between infants across the time points; PMA was used to provide a consistent measure of the infants’ developmental stage. In an analysis of the full cohort of 58 infants, we found that the microbiota of infants with growth failure had persistently low α-diversity relative to infants with appropriate growth, as measured by the Shannon Index (study weeks 1–9, p = 0.002; 30.7–44.7 weeks’ PMA, p < 0.001, Fig. 1a)^23^. This finding was consistent when we repeated the analysis including only the 41 infants who did not experience sepsis, necrotizing enterocolitis, or intestinal perforation (weeks 2–7, p < 0.001; 31.4–37.4 weeks’ PMA, p = 0.002).

Microbial community composition varied between and within individual infants over time (Fig. S1A,B). Differences between infants with appropriate growth and infants with growth failure accounted for a minor portion of the overall variation in community composition. We examined the relative abundance of bacterial taxa in the microbiota among all 58 infants in the study cohort. The representation of multiple taxa differed between infants with growth failure and appropriate growth. The microbiota of infants with growth failure had greater relative abundance of *Staphylococcaceae* in the early study weeks, followed by a persistent dominance of *Enterobacteriaceae* over the later weeks (Fig. 1b,c; Table S2). At the genus level, the microbiota of infants with growth failure had greater relative abundance of multiple *Enterobacteriaceae* members, including *Citrobacter*, *Enterobacter*, *Serratia*, *Klebsiella*, and others (Fig. 1d; Table S3). The microbiota of infants with appropriate growth had greater abundance of *Veillonellaceae* over study weeks 2–9, as well as *Streptococcaceae*, *Peptostreptococcaceae*, *Micrococcaceae*, *Lachnospiraceae*, and *Bacillaceae* over various intervals (Fig. 1b,c; Table S2). The taxonomic differences between groups were similar when only infants without sepsis, necrotizing enterocolitis, and intestinal perforation were included in the analysis (Tables S2, S3), as well as when PMA was used as the time variable in place of study week (Table S4). Many genera had a greater relative abundance in the appropriate growth group at an early PMA, followed by a greater abundance in the growth failure group at later time points, potentially indicating delayed microbiota maturation in growth failure (e.g., *Enterococcaceae unclassified*, *Escherichia*, *Pseudocitrobacter*, *Erwinia*, *Cedaceae*, *Aquimonas*, *Finegoldia*).

Recognizing that growth is dynamic over the course of the NICU stay, we also investigated microbiota features associated with catch-up growth, defined as a positive change in weight z-scores between consecutive samples, among all 58 study infants regardless of their growth outcome. Samples obtained during periods of catch-up growth (n = 84) had greater relative abundance of *Streptococcus* and many anaerobic taxa including *Bifidobacterium*, *Clostridiaceae*, *Clostridiales*, *Lachnospiraceae*, *Peptostreptococcaceae*, *Veillonella*; lower abundance of *Staphylococcus*; and higher diversity (Shannon Index, p < 0.001) than infants with a negative or neutral change in weight z-scores (Fig. S2A,B). We examined the association between catch-up growth and the change in relative abundance of the top growth-discriminatory taxa. Infants who had no change or a negative change in the relative abundance of *Veillonella* or *Streptococcus* between consecutive weeks had significantly greater reductions in weight z-scores between weeks than infants who had an increase in the abundance of these taxa (p = 0.03 for both; Fig. S2C). Collectively, the microbiota analyses indicate that growth failure is associated with low microbial diversity, a paucity of *Streptococcus* and multiple strictly anaerobic taxa, and an enrichment of *Staphylococcus* and *Enterobacteriaceae*.

### Disrupted maturation of the microbiota in growth failure

**Figure 2:**
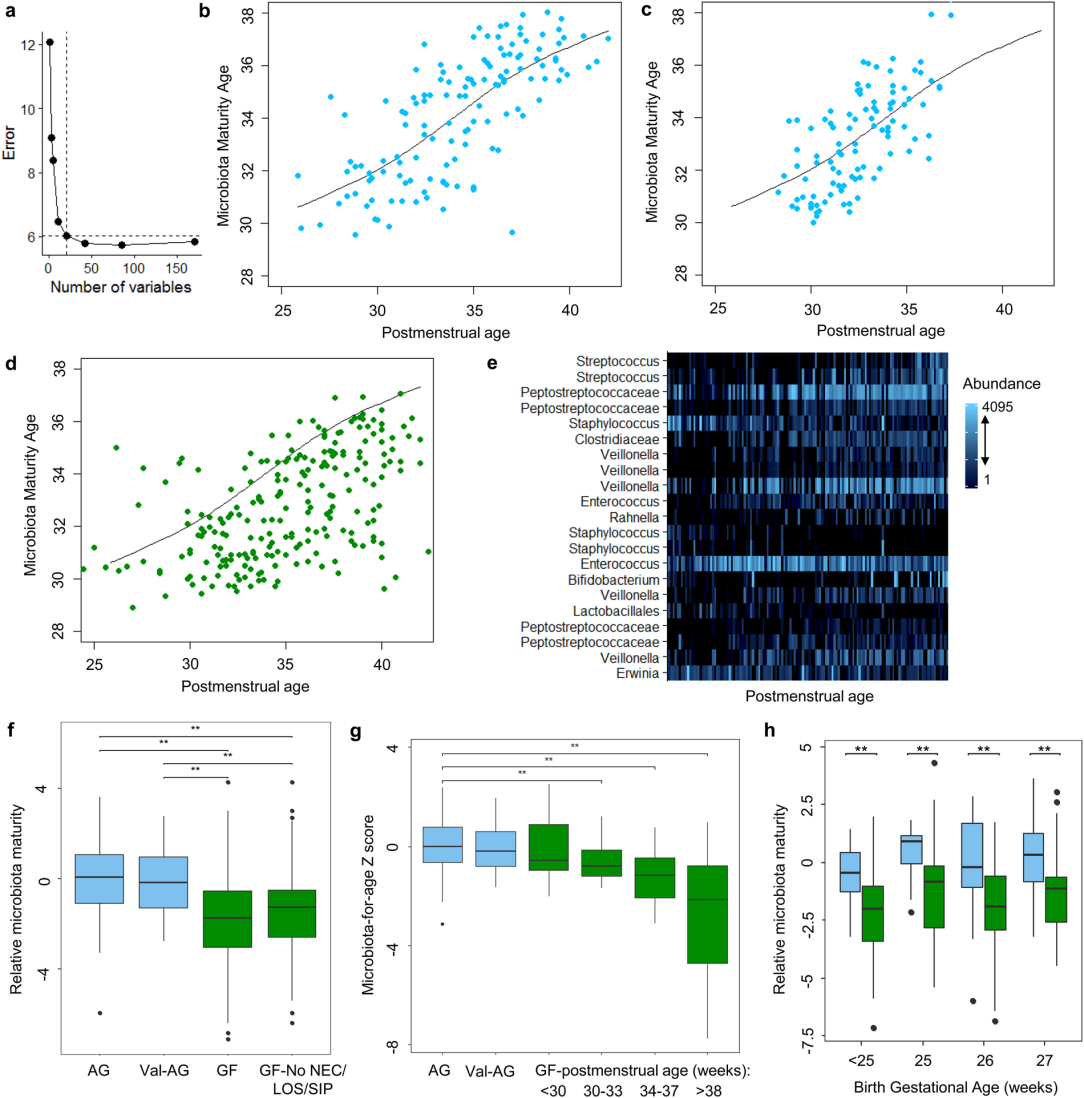
Maturation of the microbiota. A 21-feature random forest regression model was constructed with the appropriate growth samples (R^2^ = 58%, p = 0.001). The number of features was selected by cross-validation (**a**). The predicted microbiota maturity age increased with postmenstrual age (**b**). The model was applied to a separate cohort of infants with appropriate growth (**c**) and to infants with growth failure (**d**). The spline derived from the appropriate growth infants in the primary cohort is shown in each panel (**b**–**d**). The 21 features in the model and their abundance (rarefied counts) over time (*i*.*e*., postmenstrual age) are shown (**e**). Relative microbiota maturity and microbiota-for-age Z scores were similar in the two appropriate growth cohorts, but significantly lower in growth failure (**f**,**g**). Microbiota maturity was also significantly lower when the analysis was restricted to infants without sepsis, necrotizing enterocolitis, or intestinal perforation (**f**). Relative microbiota maturity age was lower in infants with growth failure than infants with appropriate growth when infants were stratified by gestational age at birth (**h**). **p < 0.05 by pairwise Wilcoxon rank sum test with Benjamini-Hochberg adjustment. AG, appropriate growth. GF, growth failure. LOS, late-onset sepsis. NEC, necrotizing enterocolitis. SIP, spontaneous intestinal perforation. Val-AG, validation cohort appropriate growth.

Given the apparent differences in microbiota composition and diversity over time, we next sought to compare microbiota maturation between infants with growth failure and appropriate growth across the complete 58 infant cohort. Random forest regression was used to model maturation of the microbiota among infants with appropriate growth following the approach of Subramanian, *et al*.^13^. Rarefied operational taxonomic unit (OTU) counts were regressed against the infant’s PMA at the time of sampling. The top age-discriminatory OTUs were ranked in order of their contribution to model accuracy and cross-validation was used for feature selection. A total of 21 OTUs were retained in a sparse model, as increasing the number of variables above 21 had little impact on model error (Fig. 2a). The resulting model fit was significant when compared to a null distribution based on 1000 permutations of PMA (p = 0.001). The microbiota age predicted by the model was plotted against PMA and a smoothing spline was fit (Fig. 2b). The 21-feature model was then applied to a separate cohort of 15 preterm infants with appropriate growth (median birth gestational age 28 [range: 25–31] weeks) and a similar rise in microbiota maturity age was seen with PMA (Fig. 2c). Last, the model was applied to the samples from infants with growth failure (Fig. 2d). The predicted microbiota maturity ages of most of the growth failure samples fell below the curve derived from the infants with appropriate growth, suggesting that the infants with growth failure had delayed or disrupted maturation of the microbiota. The taxa in the model included *Streptococcus*, *Peptostreptococcaceae*, *Veillonella*, and others (Fig. 2e). Relative microbiota maturity and microbiota-for-age Z (MAZ) scores were significantly lower among infants with growth failure than infants with appropriate growth in the primary and validation cohorts (p < 0.001 for both; Fig. 2f,g). Microbiota maturity age remained significantly lower among the infants with growth failure after excluding infants with sepsis, necrotizing enterocolitis, and intestinal perforation (Fig. 2f). Given the potential influence of birth gestational age on microbiota maturation^17^, we compared relative microbiota maturity between infants with growth failure and infants with appropriate growth stratified by birth gestational age (*i*.*e*., completed weeks of gestation at birth). Relative microbiota maturity age was significantly lower in growth failure regardless of birth gestational age (Fig. 2h).

### Postnatal growth failure is associated with altered metabolism

We next compared serum metabolomic profiles of infants with growth failure and appropriate growth, including all 58 infants in the cohort. Flow-injection, targeted, tandem mass spectrometry (FI-MS/MS) was used to measure a panel of amino acids and acylcarnitines, followed by further characterization of acylcarnitines by liquid chromatography-tandem mass spectrometry (LC-MS/MS). Gas chromatography-mass spectrometry (GC-MS) was used for non-targeted metabolomic analysis. No significant differences were observed between the two groups in the first postnatal week (study week 0) by univariate analyses of individual metabolites or by multivariate partial least squares-discriminant analysis of the combined targeted amino acids, acylcarnitines, and non-targeted metabolites. Metabolomic profiles clustered by postnatal day at sampling, primarily driven by shifts in acylcarnitine concentrations (Fig. S3A,B), likely reflecting maturation and nutritional changes in the first postnatal week (Table S1).

Next, we compared metabolomic profiles between the two growth groups over time in the full 58 infant cohort, including samples that were collected while the infants were receiving full enteral nutrition. We performed principal components analysis on the combined amino acid, acylcarnitine, and metabolites measured by non-targeted GC/MS to visualize the relationships between the infants with growth failure and infants with appropriate growth. We noted variation between the metabolic profiles over time and between the two groups (Fig. S3C). We then compared individual metabolites between infants with growth failure and appropriate growth over time. There were modest differences in amino acid concentrations over various intervals, including higher concentrations of glutamine/glutamic acid and proline in infants with growth failure and higher concentrations of methionine and histidine in infants with appropriate growth, but these differences were not significant in the analysis of infants without sepsis, necrotizing enterocolitis, or intestinal perforation (Table S5).

We next examined acylcarnitine concentrations in all study infants. The concentration of most acylcarnitines decreased over time, but there were persistent elevations in a number of short- and medium-chain acylcarnitines in the growth failure group relative to the appropriate growth group (Fig. S4A,B). Concentrations of certain long-chain acylcarnitines (*e*.*g*., C18:1, C18:2, C20:4) were higher in the appropriate growth group (Fig. S4B). These findings were similar when the analysis was restricted to infants without sepsis, necrotizing enterocolitis, or intestinal perforation, with higher concentrations of multiple medium-chain acylcarnitines in the growth failure group, and higher long-chain acylcarnitines in the infants with appropriate growth (Table S6). Similar temporal changes and relationships between groups were observed in the larger set of acylcarnitines measured by LC-MS/MS (Fig. S4C).

In the non-targeted GC-MS metabolomic analysis of the full 58 infant cohort, infants with growth failure had higher levels of multiple fatty acids and their products of oxidation, including palmitoleic acid, lauric acid, the fatty acid-derived ketone body β-hydroxybutyric acid, and the diacidic fatty acid, azelaic acid, which can be formed during ω*-*oxidation (Fig. S5A). The findings were similar in the cohort of infants without sepsis, necrotizing enterocolitis, or intestinal perforation (Fig. S5B). These changes were accompanied by increases in glycerol, a byproduct of lipolysis in adipose tissue. Overall, these results suggest that infants with growth failure have altered metabolic development relative to infants with appropriate growth, particularly related to lipolysis and fatty acid oxidation.

### Disrupted maturation of the metabolome in growth failure

**Figure 3:**
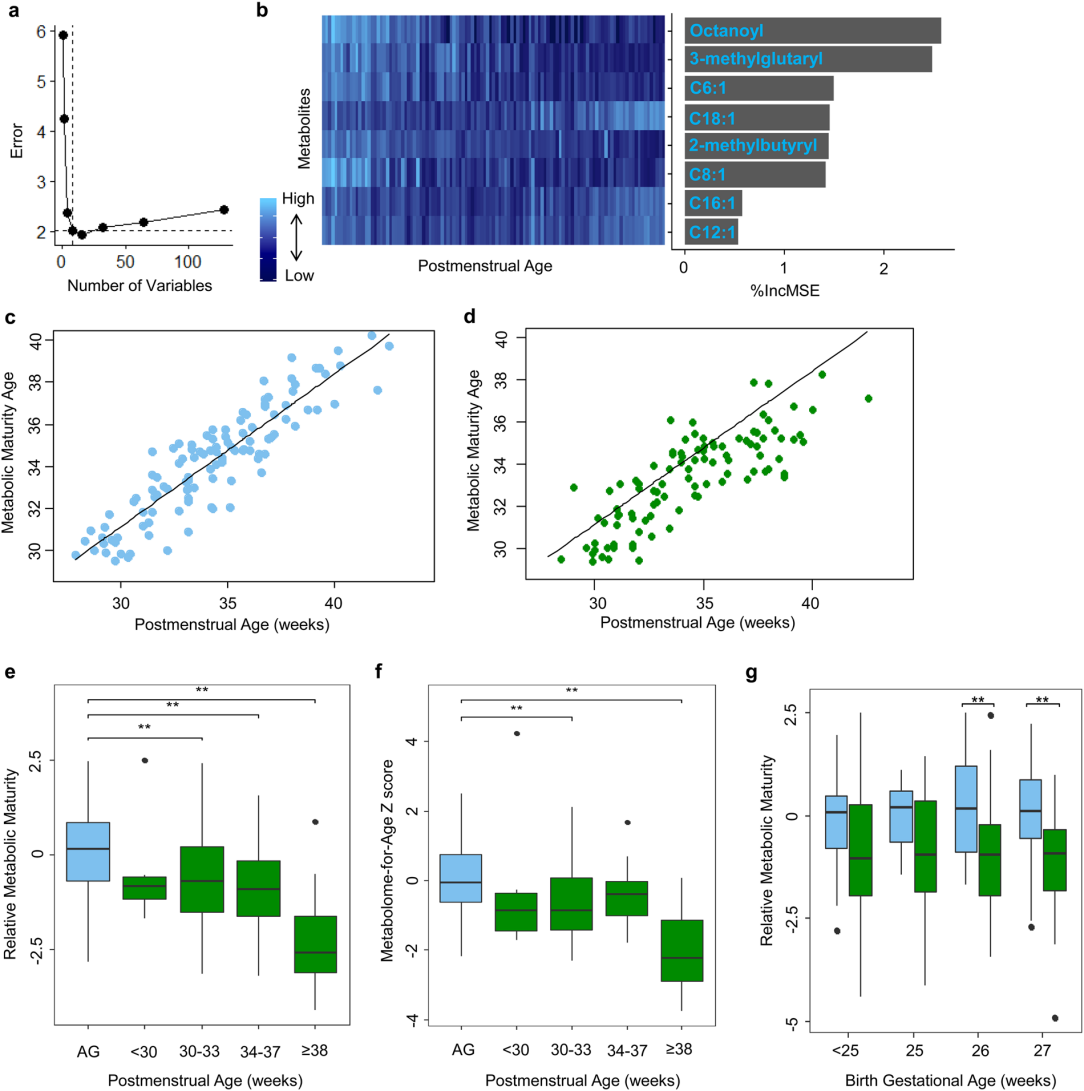
Maturation of the metabolome. An 8-feature random forest regression model was constructed using acylcarnitine profiles of infants with appropriate growth. (**a**) The number of features was selected by cross-validation. (**b**) Heatmap of the 8 metabolites included in the model over time, ranked in order of importance in the model by the percent difference in mean squared error (%IncMSE). (**c**) Metabolic maturity age increased with postmenstrual age in the appropriate growth infants. (**d**) The model was then applied to infants with growth failure. The metabolic maturity age of many of the infants with growth failure fell below the spline derived from infants with appropriate growth in both the primary analysis of the full 58 infant cohort and the secondary analysis of infants without sepsis, necrotizing enterocolitis, or intestinal perforation (shown). (**e**) Relative metabolic maturity and metabolome-for-age Z scores were significantly lower in growth failure (green) than in appropriate growth (blue), both when including all infants and when including only infants without sepsis, necrotizing enterocolitis, or intestinal perforation (shown). (**g**) Relative metabolic maturity was lower among infants with growth failure (green) than infants with appropriate growth across birth gestational age strata (blue); the difference between groups was statistically significant among infants born at 26 weeks and 27 weeks of gestation. **p < 0.05, as determined by the pairwise Wilcoxon rank sum tests with Benjamini-Hochberg adjustment. AG, appropriate growth.

Given the apparent differences in metabolic development between infants with growth failure and infants with appropriate growth, we used the same random forest regression approach as described in the microbiota analysis, but with metabolites in place of microbial taxa, to model maturation of the metabolome in infants with appropriate growth. The resulting model explained 80% of the variation in relation to postmenstrual age (p = 0.004). We retained the top 8 features in a reduced model based on cross-validated prediction performance of the model with sequentially fewer variables (Fig. 3a). The top features in the appropriate growth model included octanoyl carnitine, 3-methylglutaryl carnitine, hexenoyl (C6:1) carnitine, 2-methylbutyrl carnitine, and octenoyl (C8:1) carnitine, which decreased over time, and octadecenoyl (C18:1) carnitine, hexadecenoyl (C16:1) carnitine, and dodecenoyl (C12:1) carnitine, which increased over time (Fig. 3b). The reduced 8-feature model was applied to the appropriate growth infants (R^2^ = 80%, p = 0.005) and a smoothing spline was fit (Fig. 3c). The model was then applied to the infants with growth failure, and the relative metabolic maturity was compared to the spline derived from the infants with appropriate growth (Fig. 3d). Infants with growth failure had significantly lower relative metabolic maturity and metabolome-for-age Z scores (p < 0.001), indicating that growth failure is associated with delayed metabolic maturation. Metabolic maturation was also significantly delayed in growth failure in the cohort of infants without sepsis, necrotizing enterocolitis, or intestinal perforation (Fig. 3d–f). We also examined the relationship between metabolic maturation and birth gestational age. Relative metabolic maturity did not differ significantly between birth gestational age strata (*i*.*e*., completed weeks of gestation at birth). Median relative metabolic maturity was lower among infants with growth failure than infants with appropriate growth when compared within birth gestational age groups (Fig. 3g).

### Relationships between the microbiota and metabolome

**Figure 4:**
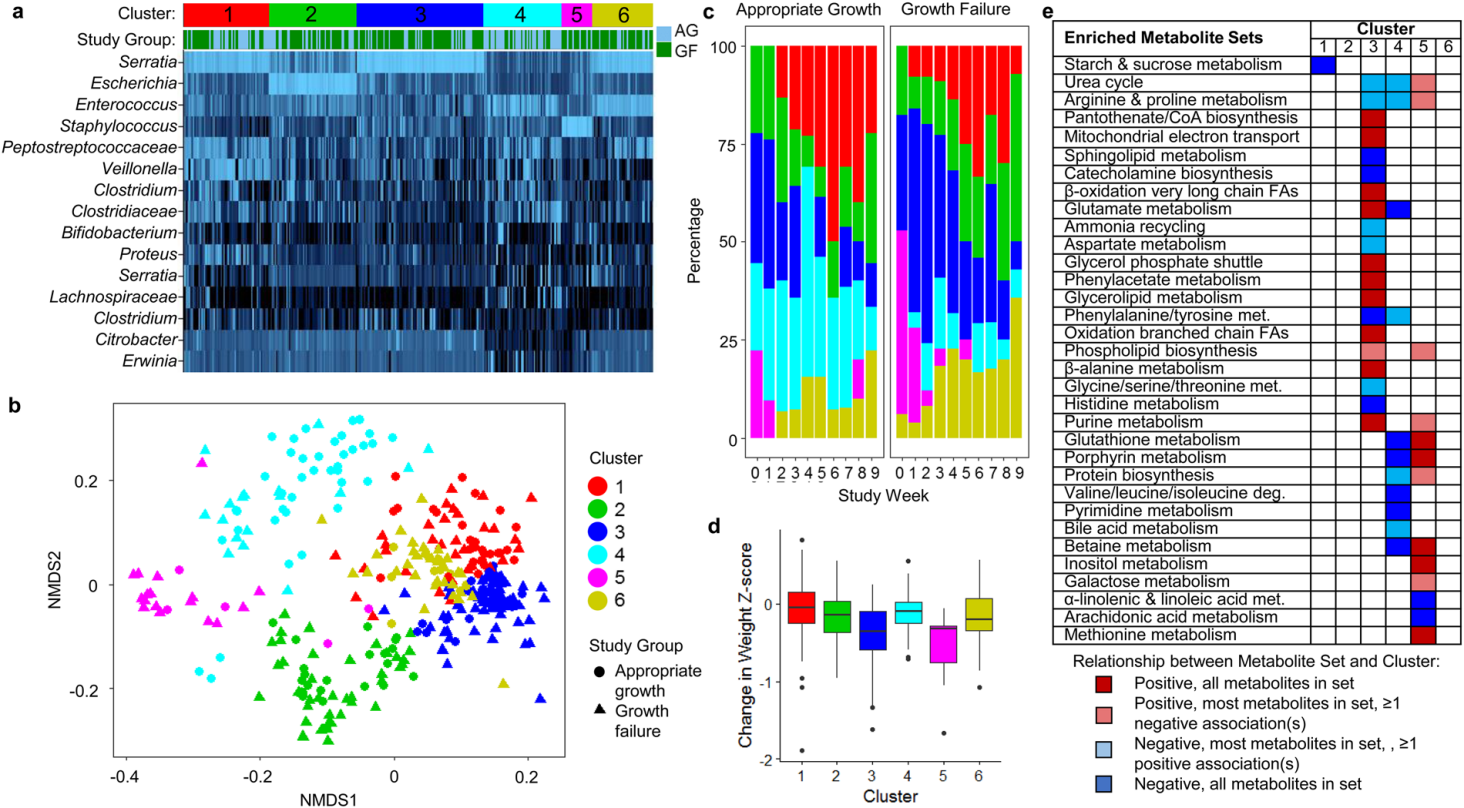
Microbiota clusters. (**a**) Relative abundance of the top 15 OTUs in the 6 clusters. (**b**) Non-metric multidimensional scaling (NMDS) plot of clusters. (**c**) The distribution of clusters by growth group and time. (**d**) Change in weight z-scores between consecutive samples. Clusters 3 and 5 were associated with significantly greater weight z-score reductions than samples in clusters 1, 2, 4, and 6. (**e**) Metabolite sets enriched in clusters. AG, appropriate growth; deg., degradation; FAs, fatty acids; GF, growth failure; met., metabolism.

To elucidate relationships between microbial communities and metabolic functions, we used partitioning around medoid (PAM) clustering to group all microbiota samples into 6 clusters (Fig. 4a,b). The distribution of clusters differed between growth groups and with time (Fig. 4c). At later time points, samples were more likely to be included in Clusters 1, 2, and 6, and less likely to fall into Clusters 3 and 5 (p < 0.01 for all). Growth failure was associated with Cluster 3 (p = 0.02). Clusters 3 and 5 were associated with greater reductions in weight z-scores between weeks than Clusters 1, 2, 4, and 6 (Fig. 4d). The microbiota samples were paired with the corresponding metabolomic samples that were collected from the same individual and time point, and metabolite set enrichment analysis was used to identify metabolite sets that were enriched in each cluster (Fig. 4e). The metabolomic profiles of samples linked to the samples in the growth failure-associated Cluster 3 were enriched in several lipid metabolism pathways including fatty acid β-oxidation, glycerolipid metabolism, phospholipid biosynthesis, and branched-chain fatty acid oxidation.

We explored the relationship between Cluster 3 with its nearest neighbors Clusters 1 and 6 (Fig. 4b). All three clusters were enriched in a common *Serratia* OTU, but Cluster 3 was associated with poor growth relative to Clusters 1 and 6, suggesting that the poor growth may be related to a paucity of taxa enriched in Clusters 1 and 6 rather than the *Serratia* dominance. Taxa co-enriched in Clusters 1 and 6 relative to Cluster 3 included *Veillonella* (Cluster 1) and *Peptostreptococcaceae* (Clusters 1 and 6). We explored correlations between *Veillonella* and *Peptostreptococcaceae* OTUs and individual host metabolites. These taxa were positively correlated with certain amino acids and amino acid metabolites (*e*.*g*., *Veillonella* with histidine; *Peptostreptococcaceae* with citrulline and indoleacetic acid) and negatively correlated with indolelactic acid and multiple acylcarnitines (Fig. S6).

## Discussion

We found that EPT infants with postnatal growth failure had disrupted maturation of the microbiota characterized by low diversity, persistent dominance of *Enterobacteriaceae*, and a paucity of strictly anaerobic taxa including *Veillonella* compared to infants with appropriate growth. Further, the infants with growth failure demonstrated a metabolic signature of increased lipolysis and fatty acid oxidation, characterized by elevations in multiple fatty acids, acylcarnitines, glycerol, and β-hydroxybutyric acid. Under normal physiological conditions, fatty acid mobilization and oxidation are enhanced in the fasted compared to the fed state. Malnourished children in Uganda present with a similar profile of elevated fatty acids, acylcarnitines and ketone metabolites, all of which are lowered by a two-week period of nutritional rescue^24^. In contrast, the infants with growth failure in our study had a persistent physiologic state resembling fasting despite similar caloric intake as the infants with appropriate growth. This may suggest that infants with growth failure have deficiencies in anabolic metabolism of glucose and other non-lipid fuels, leading to a greater reliance on fatty acids to meet metabolic demands. Interestingly, transplantation of microbial communities from malnourished children in Malawi into germ-free mice results in elevated levels of multiple acylcarnitine species relative to mice transplanted with the same microbiome supplemented with a 5-member microbial community from healthy children^14^. These findings demonstrate that the microbiome of malnourished children can impart a metabolic phenotype indicative of enhanced fatty acid mobilization and oxidation, similar to that reported here. The enrichment of key pathways that are expected to contribute to the metabolite signature of lipid mobilization and oxidation (*e*.*g*., fatty acid oxidation pathways and glycerolipid metabolism) in the growth failure-associated Cluster 3 suggests that the unique composition of bacterial communities in growth failure might contribute to a metabolic state with similarities to fasting. Our finding of impaired metabolic maturity in growth failure is consistent with this concept.

The etiology of growth failure in extremely preterm infants is complex and multifactorial. The persistent differences in the microbiota and metabolome that preceded growth failure in the subgroup of infants without major infectious and intra-abdominal complications indicates that the observed differences are not solely attributable to these morbidities. However, we cannot exclude the possibility that other host or clinical confounding factors contributed to the observed associations between the microbiota, metabolome, and growth. Studies in other model systems indicate that the early microbiota directly influences postnatal growth^20,25,26^. A recent study examined the effects of the preterm infant microbiota on intestinal maturation and gene expression in germ-free mice^18,19^. The mice were colonized with the microbiota of two preterm infants, one of whom had slower weight gain than the other. Recipients of the microbiota from the infant with slower growth had increased NF-kB activation, decreased markers of intestinal epithelial maturation, and diminished growth compared to the mice that received the microbiota of the infant with a higher growth rate. While the study was limited to two infants, the results suggest that the preterm infant microbiota may directly modulate intestinal development and postnatal growth.

Other studies provide testable models of how the altered microbiota in growth failure may modulate metabolic maturity. Studies in gnotobiotic models have shown that the microbiota alters both nutrient extraction and host genes involved in regulating energy expenditure, including fatty acid oxidation^27,28^. Differences in the functions of the microbiota and the production of bioactive metabolites by the microbiota in growth failure may have effects on host metabolism, as microbial metabolites such as the short-chain fatty acids have a role in regulating glucose homeostasis and lipid metabolism^29,30^. Differences in innate immune responses to the altered microbiota in growth failure represent another potential mediator of the microbiota-metabolome associations observed in our study. For example, the high burden of *Enterobacteriaceae* in growth failure may have led to increased inflammation through toll-like receptor 4 activation, which could in turn decrease insulin sensitivity and increase lipolysis markers, since insulin suppresses lipolysis in adipose tissue^31^. The finding of a low diversity microbiota in the infants with growth failure could also contribute to impaired growth, as low microbial diversity is associated with impaired barrier function and intestinal inflammatory conditions including necrotizing enterocolitis in preterm infants^32,33^.

Two recent observational studies investigated the microbiota and preterm infant growth. Arboleya, *et al*. examined the abundance of 8 bacterial taxa in the first month of life and weight gain in infants born at 28–33 weeks of gestation. Some of the same taxa were negatively associated with growth as in our study, including *Staphylococcus* and *Enterobacteriaceae*^34^. Grier, *et al*. grouped the microbiota of a cohort of preterm and full term infants into three phases^35^. Phase 2, which was enriched in *Enterobacteriaceae*, was associated with prematurity and negatively associated with weight z-scores in a multivariate regression model. The integrated analysis of the fecal microbiota and serum metabolome in our current study adds new insight into changes in host metabolism that accompany the altered microbiota in growth failure.

Additional study is needed to reproduce our findings in independent cohorts to determine if these signatures of microbiota and metabolomic maturation apply broadly to infants with and without growth failure. Current practice is limited by a lack of biomarkers to identify the infants at highest risk of growth failure, guide personalized interventions, and measure the response to therapy. Simply increasing caloric supply in growth failure without understanding the infant’s capacity to effectively utilize the added nutrients is often ineffective and may have unintended consequences, such as the accumulation of metabolites to levels that are toxic to developing organs, diversion of nutrients to fuel concomitant inflammatory processes, or the promotion of adiposity relative to lean body mass. Understanding how nutritional efficacy is altered by the infant microbiota and metabolic maturity may yield novel approaches to predict, prevent, and treat growth failure in preterm infants.

## Methods

### Study design

We enrolled EPT infants in the Duke NICU, excluding infants with major congenital anomalies, abdominal surgery prior to enrollment, or small-for-gestational age (birth weight <10^th^ percentile). Severe postnatal growth failure was defined as weight <3^rd^ percentile on Fenton growth charts at 40 weeks’ PMA, or at hospital discharge for infants discharged prior to 40 weeks’ PMA. The study was approved by the Duke Institutional Review Board and carried out in accordance with relevant guidelines and regulations. Written informed consent was obtained from parents. Stool was collected from diapers. Serum samples were scavenged from blood that was collected for routine laboratory testing. Samples were stored at −80 °C until analysis.

### Microbiome analysis

Genomic DNA was extracted from fecal samples using bead-beating and extraction kits (Zymo Research). PCR was performed using primers targeting the V4 region (515F/806R) of the 16S rRNA gene^36^. PCR amplicons were pooled and sequenced on the Illumina MiSeq platform. Sequences were filtered, trimmed, and paired ends were overlapped using QIIME scripts^37^, resulting in a median of 42,546 (IQR: 30464, 65609) high quality reads per sample. Reads with >97% shared sequence identity were clustered into OTUs. Taxonomy assignments were made by aligning representative sequences to the SILVA database^38^. OTUs in *Enterobacteriaceae* family that did not have a lower taxonomic assignment were further classified using the RDP classifier^39^. Sparse OTUs that did not have counts of more than 10 in at least 10% of samples and samples with <50 filtered reads were removed. A total of 385 samples were analyzed, including a median of 6 (IQR: 4, 8) samples per subject in the cohort of infants without sepsis, necrotizing enterocolitis, or intestinal perforation.

### Metabolomic analysis

Targeted FI-MS/MS was used to quantify a panel of 45 acylcarnitines and 15 amino acids in the infant serum using stable isotope dilutions^11,40^. We removed C7:DC, which had a concentration below the limit of detection in >25% of the samples. Acylcarnitines were further characterized by LC-MS/MS^41,42^. Samples in which <50% of metabolites were detected and metabolites that were undetected in >25% of samples were removed. Zero values were replaced by half of the minimum value for each metabolite. Concentrations were then log-2 transformed and Pareto scaled in MetaboAnalyst 3.0^43^. The median number of blood samples per subject was 8 (IQR: 6, 9).

Samples with sufficient remaining volume were analyzed by non-targeted GC-MS^44,45^. Quality controls were included in each sample batch. Annotations were assigned using an internal GC-MS spectral library. A total of 167 features were detected, after removal of 50 features that were considered contaminants or had uncertain annotations. The dataset was filtered to remove metabolites that were included in the targeted data, not detected in all batches, or detected in <50% of samples, leaving 70 metabolites. Peak intensities were log-2 transformed and mean centered across five batch groups. Samples in which <50% of metabolites were detected were removed. The remaining missing values were imputed using K-nearest neighbor and metabolite values were Pareto scaled in MetaboAnalyst.

### Statistical analysis

#### Microbiome analysis

We determined the composition and diversity of the microbiota using functions in the phyloseq package^46^. We used smoothing spline analysis of variance (SS-ANOVA) models with 1000 permutations for between-group comparisons over time^47–49^. Sequencing counts were normalized by cumulative sums scaling^47^. The relative abundance of bacterial taxa at the family and genus level were compared between groups using the fitTimeSeries function in the metagenomeSeq package^49^. The relationships between microbiota community structure in infants with growth failure and appropriate growth were examined using principal coordinates analysis (PCoA) on Jensen-Shannon divergences (JSD).

Samples from the appropriate growth group were used to construct a model of microbiota maturation^13^. The trimmed OTU table from the primary cohort was used as a closed reference for OTU picking in a separate validation cohort of preterm infants with appropriate growth. Seven OTUs that were present in the primary cohort but not the validation cohort were removed. OTU counts were rarefied to 5000 counts. The OTU counts from the appropriate growth infants in the primary cohort were regressed against infant PMA at the time of sampling by random forest regression with 500 trees. Features were ranked in order of importance and 100-fold cross validation was used to select the number of features to retain with the rfcv function in the randomForest package^50^. We then constructed a sparse model using only the retained features that accounted for the greatest marginal difference in mean squared error. The significance of the model fit was determined by comparing the results to a null distribution using 1,000 random permutations of infant PMA. We plotted the predicted microbiota age by PMA for each sample and fit a smoothing spline. The sparse model was then applied to the infants in the validation cohort and the infants with growth failure. The results were compared to the spline derived from the appropriate growth infants. Relative microbiota maturity was defined as the predicted microbiota age of the infant minus the microbiota age of infants with appropriate growth at the same PMA, as determined by the smoothing spline^13^. MAZ scores were calculated by subtracting the median microbiota age of PMA-matched appropriate growth infants from the predicted microbiota age, and dividing the result by the standard deviation of the microbiota age of PMA-matched appropriate growth infants. For this analysis, samples were grouped into four one-month intervals (<30, 30–33, 34–37, and >38 weeks’ PMA).

#### Metabolomic analyses

Metabolomic profiles of infants with growth failure and appropriate growth were compared in study week 0 using t-tests for individual metabolites and partial least squares-discriminant analysis for metabolomic profiles. Next, temporal differences in metabolites between groups were examined using principal components analysis and SS-ANOVA. Metabolic maturation was modeled using the same random forest regression approach described in the microbiota analysis. The metabolomic datasets were combined retaining only samples with complete LC-MS/MS acylcarnitine, amino acid, and GC-MS data that were obtained from infants who were receiving complete enteral nutrition to minimize the confounding effects of parenteral nutrition on serum metabolites. Metabolite values were regressed against PMA in the appropriate growth infants. The resulting model explained 79% of the variation related to PMA (p = 0.001). We selected the top features to retain in a reduced model based on cross-validated prediction performance with sequentially fewer variables. We found that all of the top variables that contributed substantially to model performance were present in the LC-MS/MS acylcarnitine dataset, including 3-methylglutaryl, octanoyl, C18:1, 2-methylbutyryl, and C10:2 carnitine. The combined metabolomics dataset of acylcarnitines, amino acids, and metabolites identified by non-targeted GC-MS analysis contained fewer samples than the LC-MS/MS acylcarnitine dataset due to the exclusion of samples with insufficient volume for GC-MS analysis. Given that all of the top predictors in the model were acylcarnitines, we constructed a new model using only the acylcarnitine data to maximize the number of samples included. We compared relative metabolic maturity and metabolome-for-age Z scores between groups, defined by the same criteria as in the microbiota analyses. There was no validation cohort for the metabolic maturity model.

#### Relationships between the microbiota and metabolome

Microbiota samples were clustered using PAM including the most significant PCoA eigenvectors from a JSD matrix. The number of clusters was determined using the gap statistic^51^. Repeated measures logistic regression was used to determine whether time and study group were predictors of cluster, considering each cluster in a separate model as a binary outcome. Metabolomic samples were paired with their corresponding microbial samples and quantitative metabolite set enrichment analysis was performed in MetaboAnalyst 3.0. Significant metabolite sets (FDR p < 0.05) were enriched in the specified cluster in comparison to all other samples. We examined correlations between individual metabolites and *Veillonella* and *Peptostreptococcaceae* OTUs using Spearman correlation.

Statistical analyses were performed in the R environment (version 3.3.2). Data were visualized using the ggplot2 package^52^. We routinely corrected for multiple testing by the Benjamini-Hochberg method.

## Supplementary information

Supplementary Materials

## Data Availability

Sequencing data for this study are available under BioProject PRJNA544545.
