# Supplementary material for: Disrupted Maturation of the Microbiota and Metabolome among Extremely Preterm Infants with Postnatal Growth Failure
Source: Sci Rep. 2019 Jun 3;9:8167. doi: 10.1038/s41598-019-44547-y (PMC6546715; doi:10.1038/s41598-019-44547-y)
Supplement: Supplementary file 1 — Supplementary Materials [file 41598_2019_44547_MOESM1_ESM.pdf]

Supplementary Information for

Disrupted Maturation of the Microbiota and Metabolome among Extremely Preterm Infants with Postnatal Growth Failure

Noelle E. Younge*, Christopher B. Newgard, C. Michael Cotten, Ronald N. Goldberg, Michael J. Muehlbauer, James R. Bain, Robert D. Stevens, Thomas M. O’Connell, John F. Rawls, Patrick C. Seed, Patricia L. Ashley

Address correspondence to: noelle.younge@duke.edu

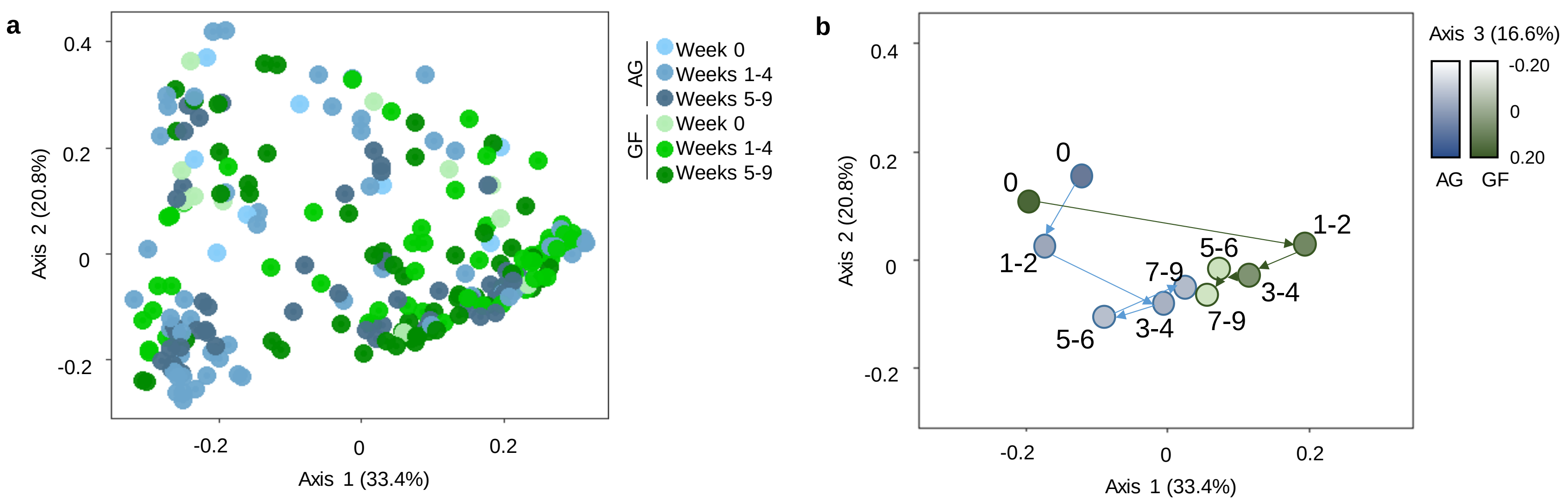

Fig. S1. Relationship between microbiota samples over time. a Principal coordinates analysis (PCoA) showing the relationship between growth groups (growth failure and appropriate growth) and variation over time based on Jenson-Shannon divergence (JSD) among infants without sepsis, necrotizing enterocolitis, or intestinal perforation. Growth group (R^2=2.7%, p=0.001), time (*i.e.*, study week; R^2=4.6%, p=0.001), and their interaction (R^2=1.5%, p=0.003) accounted for a minor portion of the overall variation in microbial community composition, as determined by permutational analysis of variance. **b** The median position of samples within each growth group and time interval (i.e., study weeks) along the first and second PCoA axes is shown to demonstrate the temporal progression. The third axis is represented by color shading. The small numbers on the plot represent study weeks. AG, appropriate growth; GF, growth failure.

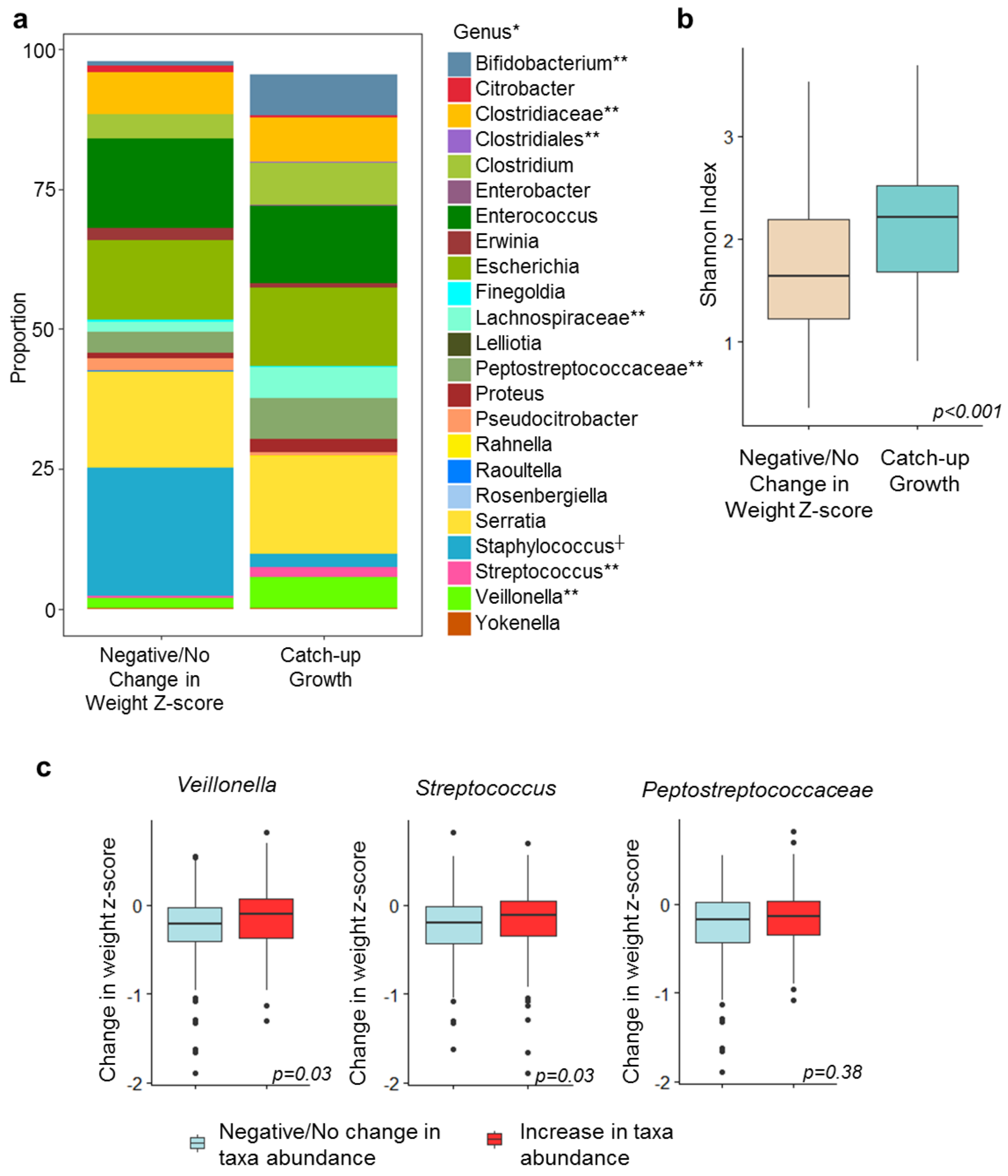

Fig. S2. Microbiota features associated with catch-up growth. **a** Relative abundance of bacterial genera among infants with neutral or negative change in weight z-scores between consecutive study weeks and infants with catch-up growth. *The lowest taxonomic assignment is given for taxa that were unassigned at the genus level. **Taxa with significantly greater relative abundance among infants with catch-up growth as determined by zero-inflated Log-normal mixture model. ┼Taxa with lower relative abundance among infants with catch-up growth. **b** Shannon Diversity Index was significantly higher among infants with catch-up growth than infants with negative/neutral change in weight z-scores ($p<0.001$ by Wilcoxon rank sum test). **c** Infants who had no change or a negative change in the relative abundance of *Veillonella* and *Streptococcus* between consecutive weeks had significantly greater reductions in weight z-scores between weeks than infants who had an increase in relative abundance of these taxa ($p=0.03$ by Wilcoxon rank sum test).

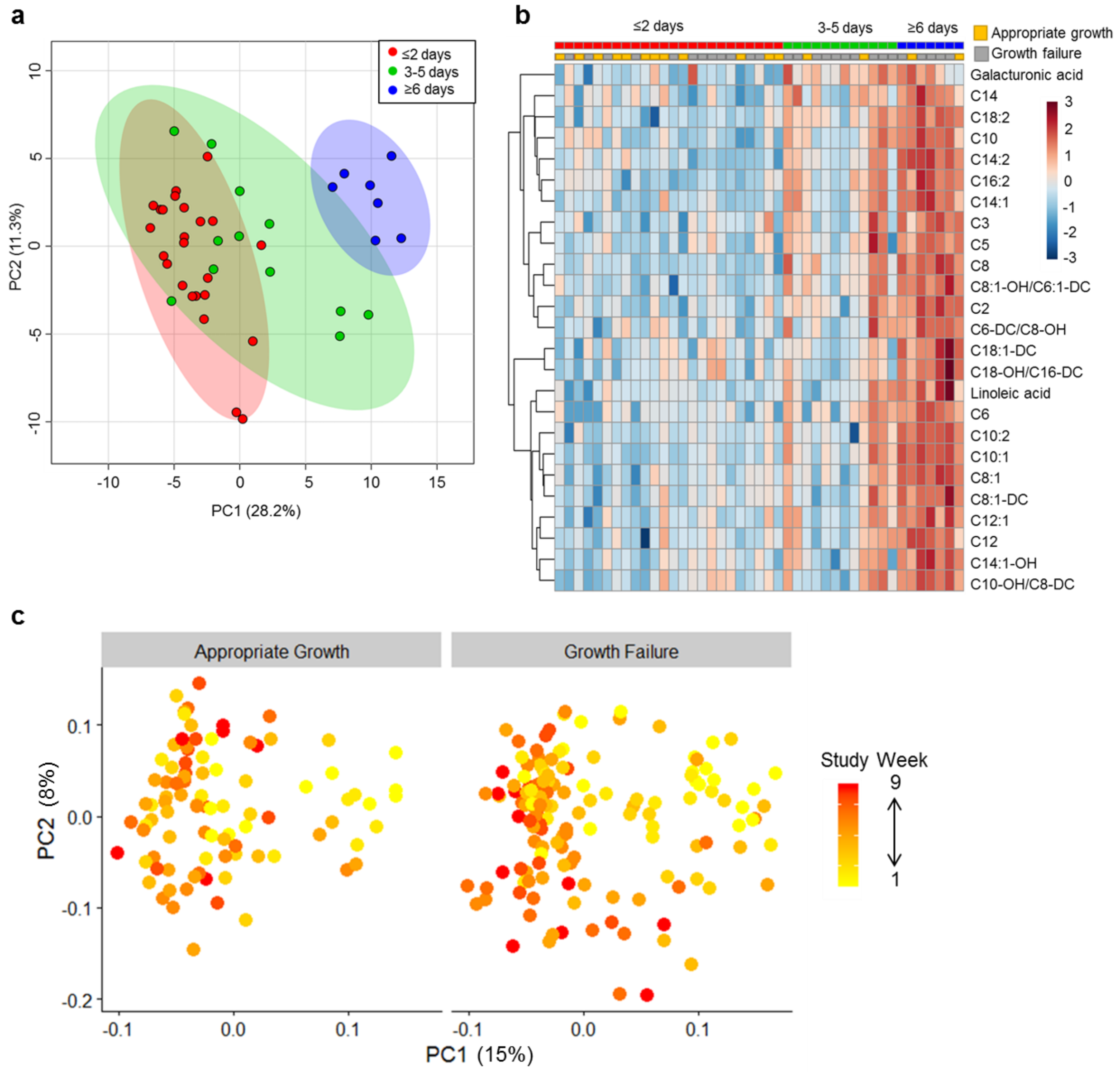

Fig. S3. Metabolomic profiles. a Principal components analysis (PCA) of metabolites in the first postnatal week in the full cohort of 58 infants. Samples clustered by time (*i.e.*, postnatal age in days). **b** Heatmap of the top 25 time-discriminatory metabolites in the first postnatal week samples. The concentration of many metabolites, particularly acylcarnitines, increased with postnatal age (in days). **c** PCA of metabolites in appropriate growth and growth failure groups over time in samples collected while the infants were receiving full enteral nutrition. The distribution of samples shifted along PC1 over time, and the median distribution of samples differed between growth groups along PC2 (Weeks 5-9, p=0.004 by SS-ANOVA).

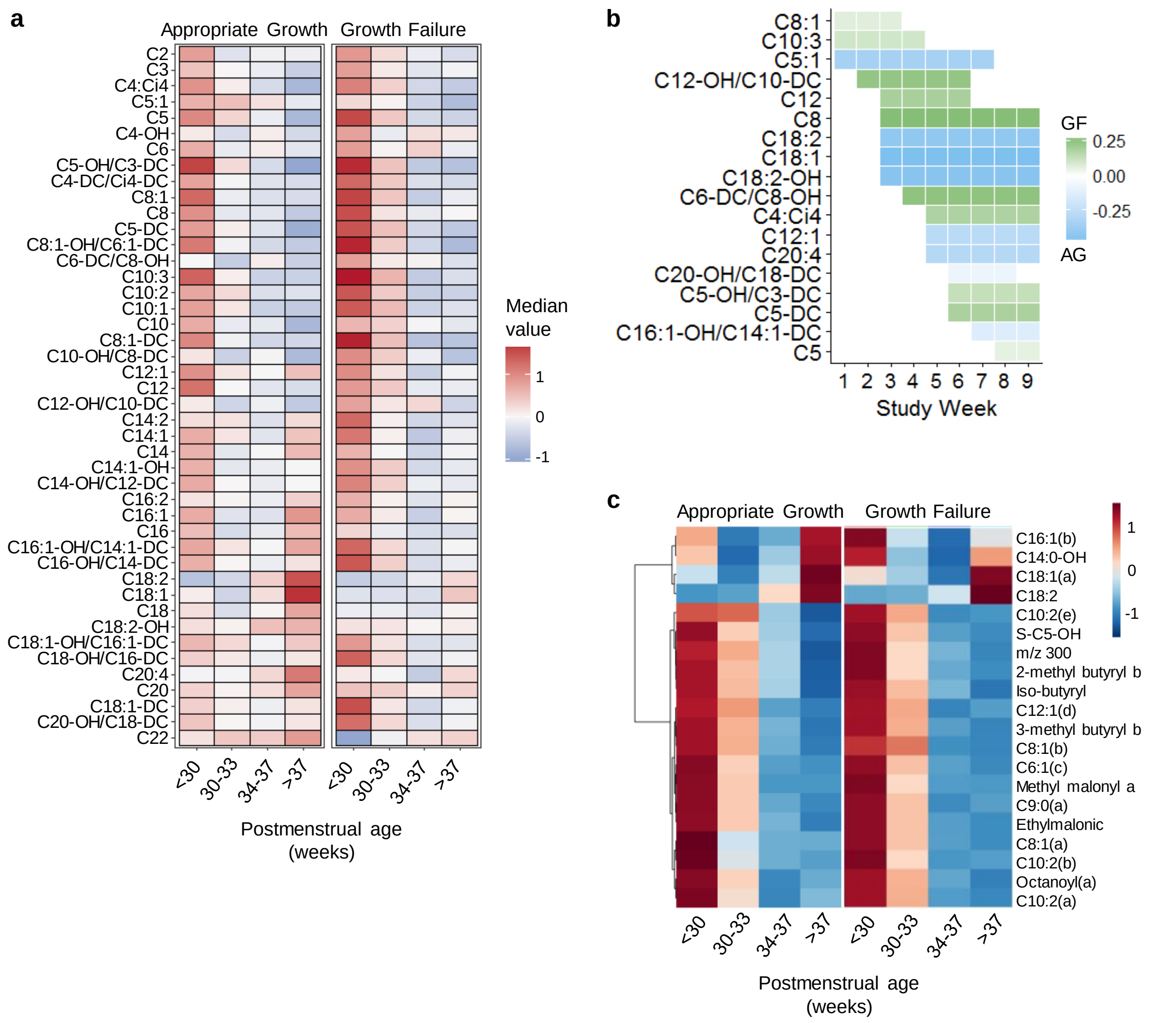

Fig. S4. Acylcarnitine profiles over time. a Heatmap showing median FI-MS/MS acylcarnitine values in samples collected while the infants were receiving full enteral nutrition in the full cohort of 58 infants. Most acylcarnitines decreased in abundance over time, but several acylcarnitines with aliphatic chains ≥18 carbons increased over time. **b** Acylcarnitines with significant differences between infants with growth failure and infants with appropriate growth by SS-ANOVA. Shading represents the time interval over which the concentration of the specified metabolite differed between groups. Green indicates greater abundance in growth failure and blue indicates greater abundance in appropriate growth. **c** Heatmap of LC-MS/MS acylcarnitine concentrations over time. The top 20 time-discriminatory metabolites in the appropriate growth infants are shown in the first panel (averaged for each time interval), and concentrations of the same metabolites are shown for the growth failure infants in the second panel. GF, growth failure. AG, appropriate growth.

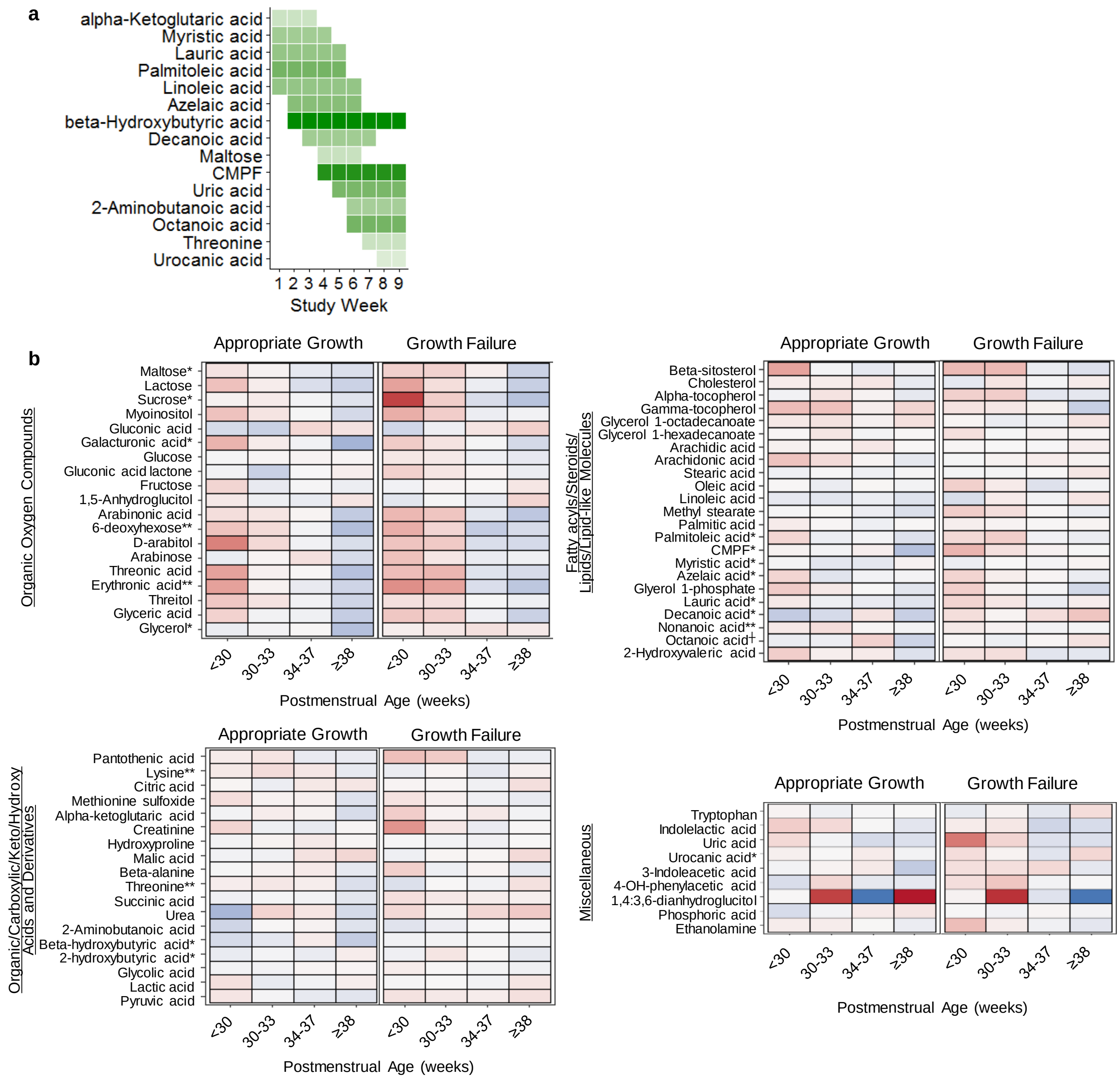

Fig. S5. Non-targeted metabolomics profiles. **a** Metabolites with significant differences between growth groups in the full cohort over time by SS-ANOVA. Shading represents the time interval over which the concentration of the specified metabolite differed between groups. All metabolites shown had greater concentration in the growth failure group (green). **b** Heatmap showing the median metabolite values for the specified time interval and growth group among infants without sepsis, necrotizing enterocolitis, or intestinal perforation, while receiving full enteral nutrition. Red shading indicates higher concentrations and blue indicates lower concentrations. Metabolites with higher concentrations in infants with growth failure (*), or infants with appropriate growth (**) are noted. Octanoic acid (†) had higher levels in appropriate growth between 30-31 weeks postmenstrual age, followed by a period with higher levels in growth failure (38-40 weeks postmenstrual age).

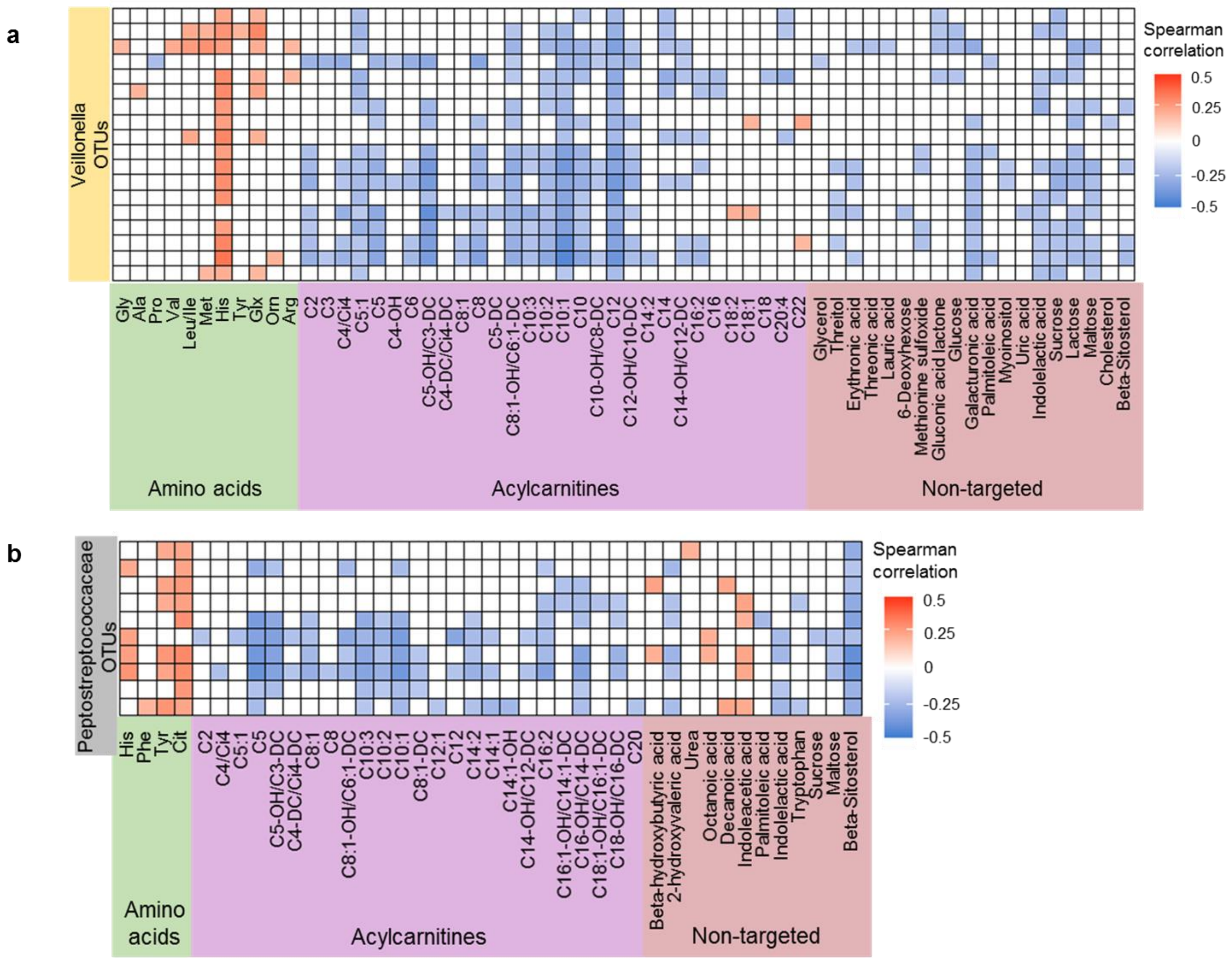

Fig. S6. Correlations between microbial taxa and metabolites. Spearman correlations between *Veillonella* (**A**) and *Peptostreptococcaceae* (**B**) OTUs and metabolites. Only significant correlations are shown ($p<0.05$ with Benjamini-Hochberg correction).

Table S1. Unit guidelines for advancement of parenteral and enteral nutrition in very low birth weight infants.

**Parenteral Nutrition**	**Enteral Nutrition**
Initiate parenteral nutrition at birth: dextrose (minimum glucose infusion rate (GIR) of 4-6 mg/kg/min) and amino acids (Trophamine) 2-3 g/kg/d	Provide oral colostrum as soon as available after birth; continue for five days.
Fat: Add fat emulsion (Intralipid 20%) at 0.5-1 g/kg/d after the first postnatal day. Advance by 0.5 g/kg daily to goal of 3.5 g/kg/d.	Initiate enteral feedings of human milk once infant demonstrates hemodynamic stability and abdominal exam is reassuring. Donor milk is used if mother's own milk is unavailable or insufficient in volume.
Dextrose: Increase by 0.5-2 mg/kg/min every day to goal of 12-15 mg/kg/min. Reduce GIR or slow the rate of advancement if infant develops hyperglycemia.	Start minimal enteral nutrition (10 mg/kg/day divided every 6 hours) for 2-5 days, then advance by 20 ml/kg/day as tolerated to goal of 150-160 ml/kg/day.
Protein: Increase by 0.5 g/kg daily to goal of 3.5-4 g/kg/d.	Add cow's milk-derived human milk fortifier once infant reaches enteral feeding volume of 80 ml/kg/day (to increase caloric content by 4 kcal/oz).
Advance macronutrients to goals of 120 kcal/kg/d and weight gain of 15-20 g/kg/day.	Add ¼ tsp of protein supplement (Beneprotein, Nestlé) per 50 ml of human milk once feeding volume reaches 150 ml/kg/day.
Standard additives include carnitine (15 mg/kg/day), pediatric multivitamins (Vitamins A, C, D, E, K, B_1, B_2, B_3, B_5, B_6, B_7, B_9, B_{12}), pediatric trace elements (chromium, copper, selenium, manganese, zinc), and heparin.	For infants with suboptimal weight gain (≤12 g/kg/day), supplement feedings with fat (Microlipid, Nestlé) and/or preterm infant formula (27-30 kcal/oz). Feeding volume may also be increased as tolerated.

Table S2. Bacterial families with significant differences in relative abundance between growth failure and appropriate growth groups over time.

	All Infants			Infants without Sepsis/NEC/SIP		
Family	Interval*	Area**	P_{adj}†	Interval*	Area**	P_{adj}†
**Higher in Growth Failure**						
Staphylococcaceae	0-5	7.89	0.029	0-4	7.03	0.032
Enterobacteriaceae	2-9	10.81	0.002	3-9	8.88	0.032
Bacteroidaceae	0-1	1.20	0.006	0-1	2.00	0.013
Erysipelotrichaceae			NS	7-9	2.37	0.020
**Higher in Appropriate Growth**						
Bacillaceae	1-6	-3.90	0.002	1-6	-4.74	0.004
Streptococcaceae	3-9	-14.22	0.002	3-9	-16.14	0.004
Peptostreptococcaceae	2-7	-10.96	0.002	3-5	-3.94	0.020
Veillonellaceae	2-9	-17.16	0.002	2-8	-20.10	0.004
Lachnospiraceae	4-7	-5.28	0.017			NS
Micrococcaceae	4-9	-3.81	0.008	5-9	-3.36	0.008
Tissierellaceae			NS	3-9	-7.88	0.019
Clostridiaceae			NS	4-8	-5.03	0.016

*Interval refers to the study weeks with significant between-group differences in the relative abundance of the specified taxa.
**Area indicates the magnitude of the difference between the observed difference and the null distribution by 1000 random permutations (i.e. a larger absolute area indicates a greater difference between groups).
†Adjusted P value by Benjamini-Hochberg correction.
NEC, necrotizing enterocolitis; NS, not significant; SIP, spontaneous intestinal perforation.

Table S3. Bacterial genera with significant differences in relative abundance between growth failure and appropriate growth groups over time.

	All Infants			Infants without Sepsis/NEC/SIP		
Genus	Interval*	Area**	P_{adj}†	Interval*	Area**	P_{adj}†
**Higher in Growth Failure**						
Enterococcaceae‡	7-9	3.17	0.037	7-9	2.17	0.023
Klebsiella	3-9	8.10	0.004	3-9	5.87	0.034
Pseudocitrobacter	4-9	7.91	0.004	5-9	6.07	0.009
Siccibacter	2-8	8.25	0.004	2-7	6.11	0.008
Leclercia	2-9	10.35	0.004	3-7	5.43	0.003
Citrobacter	0-9	13.22	0.002	0-7	10.66	0.008
Serratia	2-7	7.07	0.004	1-7	10.76	0.009
Rosenbergiella	3-9	9.91	0.004	2-8	7.03	0.003
Enterobacter	1-8	10.44	0.004	1-7	7.16	0.005
Pluralibacter	3-8	6.59	0.004	3-7	4.06	0.025
Raoultella	3-9	8.69	0.004	4-7	2.91	0.017
Budvicia	3-9	8.11	0.004			NS
Kluyvera	2-8	9.90	0.004	2-7	5.72	0.003
Yokenella	2-7	7.93	0.004	1-7	8.33	0.009
Arsenophonus	1-6	6.13	0.020	0-7	7.40	0.003
Lelliottia	0-6	7.33	0.004	0-6	6.06	0.007
Trabulsiella	1-5	5.06	0.044	0-7	8.86	0.009
Kosakonia	1-7	6.63	0.012	1-7	5.16	0.007
Morganella	0-7	9.84	0.001	0-6	8.77	0.003
Bacteroides			NS	0-1	2.03	0.005
Staphylococcus			NS	0-4	7.25	0.023
Eubacterium			NS	7-9	2.28	0.022
**Higher in Appropriate Growth**						
Clostridiaceae‡	1-9	-12.82	0.022			NS
Clostridium			NS	5-9	-7.81	0.017
Anaerobacillus	1-6	-6.75	0.002	1-6	-4.45	0.003
Streptococcus	3-9	-16.75	0.002	3-9	-15.80	0.003
Peptostreptococcaceae‡	2-7	-12.93	0.002	3-5	-3.87	0.015
Veillonella	1-9	-21.93	0.002	2-8	-19.77	0.003
Lachnospiraceae‡	5-7	-4.19	0.020			NS
Rothia	4-9	-7.09	0.004	5-9	3.16	0.005
Enterococcaceae‡	0-1	-1.44	0.038			NS
Finegoldia			NS	3-9	-7.64	0.010
Cedecea			NS	0-3	-2.41	0.003
Mangrovibacter			NS	0-3	-1.75	0.022

*Interval refers to the study weeks with significant between-group differences in the abundance of the specified taxa. **Area indicates the magnitude of the difference between the observed difference and the null distribution with 1000 random permutations.†Adjusted P value by Benjamini-Hochberg correction.‡Not classified at genus level. NEC, necrotizing enterocolitis; NS, not significant; SIP, spontaneous intestinal perforation.

Table S4. Bacterial families and genera with significant differences between infants with growth failure and infants with appropriate growth over time (postmenstrual age) among infants without sepsis, necrotizing enterocolitis, or intestinal perforation.

**Taxa**	**Interval***	**Area****	**P_{adj}†**	**Taxa**	**Interval***	**Area****	**P_{adj}†**
**Higher in growth failure**				**Higher in appropriate growth**			
**Family**							
Staphylococcaceae	24-33	19.46	0.045	*Enterococcaceae*	31-34	-4.93	0.035
Enterobacteriaceae	33-44	37.92	0.004	*Clostridiaceae*	33-44	-28.19	0.007
Tissierellaceae	40-44	12.87	0.012	*Tissierellaceae*	30-36	-9.47	0.004
Bacteroidaceae	24-29	10.11	0.035	*Bacillaceae*	30-35	-6.21	0.004
Bacteroidaceae	39-44	11.87	0.040	*Streptococcaceae*	31-44	-39.35	0.004
				Peptostreptococcaceae	32-34	-2.95	0.035
				Veillonellaceae	29-44	-51.11	0.004
				Verrucomicrobiaceae	24-31	-15.72	0.035
				Micrococcaceae	34-44	-12.83	0.012
				Clostridiales	24-28	-5.70	0.035
				Enterobacteriaceae	24-26	-4.83	0.043
**Genus**							
Staphylococcus	24-33	19.98	0.049	*Enterococcaceae*‡	28-32	-4.54	0.005
Enterococcaceae‡	36-44	23.21	0.003	*Enterococcus*	31-34	-4.82	0.027
Bacteroides	24-29	10.35	0.038	*Clostridiaceae*‡	34-44	-23.09	0.015
Finegoldia	40-44	12.78	0.018	*Finegoldia*	30-36	-9.22	0.003
Klebsiella	33-44	22.36	0.005	*Anaerobacillus*	30-35	-5.82	0.003
Pseudocitrobacter	34-44	35.70	0.003	*Pseudocitrobacter*	24-30	-15.71	0.004
Siccibacter	32-44	20.83	0.003	*Cedecea*	24-32	-9.82	0.003
Leclercia	33-44	25.55	0.003	*Raoultella*	24-25	-13.77	0.004
Citrobacter	29-44	29.55	0.004	*Mangrovibacter*	24-32	-7.09	0.021
Serratia	32-44	38.07	0.003	*Escherichia*	24-29	-13.77	0.007
Cedecea	39-44	6.03	0.014	*Aquimonas*	24-25	-1.39	0.035
Rosenbergiella	32-44	23.29	0.003	*Erwinia*	24-31	-17.22	0.003
Enterobacter	31-44	20.83	0.003	*Streptococcus*	31-44	-38.68	0.003
Escherichia	35-44	30.43	0.007	*Peptostreptococcaceae*‡	32-34	-2.87	0.031
Pluralibacter	33-44	18.81	0.003	*Veillonella*	29-44	-50.41	0.003
Raoultella	34-44	18.12	0.003	*Akkermansia*	24-31	-15.31	0.030
Aquimonas	36-44	7.15	0.003	*Rothia*	34-44	-12.15	0.004
Budvicia	34-44	16.71	0.003	*Clostridiales*	24-27	-4.28	0.033
Kluyvera	31-44	17.91	0.003	*Clostridium*	35-44	-22.87	0.025
Yokenella	30-44	21.84	0.003				
Arsenophorus	27-39	11.61	0.007				
Lelliottia	26-38	9.76	0.010				
Trabulsiella	27-28	13.84	0.011				
Kosakonia	29-41	9.16	0.004				
Erwinia	35-41	27.80	0.003				
Morganella	27-41	17.81	0.003				

*Interval refers to the weeks (postmenstrual age) with significant between-group differences in the abundance of the specified taxa.

**Area indicates the magnitude of the difference between the observed difference and the null distribution by 1000 random permutations (i.e. a larger absolute area indicates a greater difference between groups).

†Adjusted P value by Benjamini-Hochberg correction.

‡Not classified at genus level.

Table S5. Comparison of amino acids between infants with growth failure and infants with appropriate growth.*

Amino Acid	Interval (weeks)**	Area†	P_{adj}‡
**All Infants**			
**Higher in growth failure**			
Serine	8-9	0.558	0.027
Proline	5-9	1.167	0.038
Valine	8-9	0.568	0.027
Methionine	8-9	0.411	0.028
Phenylalanine	8-9	0.387	0.028
Glutamine/Glutamic acid	3-9	1.964	0.027
**Higher in appropriate growth**			
Methionine	2-4	-0.560	0.038
Histidine	2-5	-1.061	0.038
Ornithine	3-5	-0.633	0.027
Arginine	3-5	-0.635	0.038
**Infants without Sepsis/NEC/SIP**			
**Higher in growth failure**			
Glycine	2-5	0.07	0.05
Asparagine/Aspartic acid	1-5	0.17	0.05
Glutamine/Glutamic acid	1-5	0.18	0.05
**Higher in appropriate growth**			
Methionine	1-5	-0.11	0.05
Tyrosine	1-9	-0.42	0.05
Histidine	1-5	-0.17	0.05

*Infants were receiving full enteral nutrition (i.e. no parenteral nutrition) at the time of sampling.

**Interval refers to the study weeks with significant between-group differences in the concentration of the specified metabolite.

†Area indicates the relative magnitude of the difference between the observed difference and the null distribution by 1000 random permutations (i.e. a larger absolute area indicates a greater difference between groups).

‡Adjusted P value by Benjamini-Hochberg correction.

Table S6. Acylcarnitines with significant differences between infants with growth failure and infants with appropriate growth over time*

	Interval (Postmenstrual Age, weeks)**	Area†	P_{adj}‡
**Higher in growth failure**			
C5-OH/C3-DC	34-44	0.9	0.02
C8	32-44	0.78	0.03
C5-DC	37-43	0.71	0.02
C10	35-44	0.56	0.04
C12	33-37	0.21	0.04
C16:1-OH/C14:1-DC	26-29	0.02	0.04
C16-OH/C14-DC	26-33	0.45	0.03
**Higher in appropriate growth**			
C3	27-30	-0.18	0.049
C5:1	26-35	-0.91	0.03
C12:1	35-44	-0.84	0.04
C16:1	35-44	-0.91	0.04
C18:2	32-44	-1.56	0.02
C18:1	33-44	-1.42	0.02
C18:2-OH	28-38	-0.69	0.03
C20:4	35-44	-1.19	0.03
C20-OH/C18-DC	35-44	-0.92	0.04
C22	26-35	-1.31	0.04

*Only the samples that were collected while the infant was receiving all enteral nutrition (i.e. no parenteral nutrition) were included in this analysis.

**Interval refers to the weeks with significant between-group differences in the concentration of the specified metabolite.

†Area indicates the magnitude of the difference between the observed difference and the null distribution by 1000 random permutations (i.e. a larger absolute area indicates a greater difference between groups).

‡Adjusted P value by Benjamini-Hochberg correction.
